# Survey of volatile substances in kitchen utensils made from acrylonitrile–butadiene–styrene and acrylonitrile–styrene resin in Japan

**DOI:** 10.1002/fsn3.100

**Published:** 2014-03-05

**Authors:** Yutaka Abe, Miku Yamaguchi, Motoh Mutsuga, Yoko Kawamura, Hiroshi Akiyama

**Affiliations:** Division of Food Additives, National Institute of Health Sciences1-18-1 Kamiyoga, Setagaya-ku, Tokyo, 158-8501, Japan

**Keywords:** Acrylonitrile–styrene resin (AS), acrylonitrile–styrene–butadiene resin (ABS), food contact material, kitchen utensil, volatile substance

## Abstract

Residual levels of 14 volatile substances, including 1,3-butadiene, acrylonitrile, benzene, ethylbenzene, and styrene, in 30 kitchen utensils made from acrylonitrile–butadiene–styrene resin (ABS) and acrylonitrile–styrene resin (AS) such as slicers, picks, cups, and lunch boxes in Japan were simultaneously determined using headspace gas chromatography/mass spectroscopy (HS-GC/MS). The maximum residual levels in the ABS and AS samples were found to be 2000 and 2800 *μ*g/g of styrene, respectively. The residual levels of 1,3-butadiene ranged from 0.06 to 1.7 *μ*g/g in ABS, and three of 15 ABS samples exceeded the regulatory limit for this compound as established by the European Union (EU). The residual levels of acrylonitrile ranged from 0.15 to 20 *μ*g/g in ABS and from 19 to 180 *μ*g/g in AS. The levels of this substance in seven ABS and six AS samples exceeded the limit set by the U.S. Food and Drug Administration (FDA). Furthermore, the levels of acrylonitrile in three AS samples exceeded the voluntary standard established by Japanese industries. These results clearly indicate that the residual levels of some volatile compounds are still high in ABS and AS kitchen utensils and further observations are needed.

## Introduction

Various plastic materials are used in kitchen utensils such as dishes, cups, spoons, forks, measuring cups, and bottles. Acrylonitrile–butadiene–styrene resin (ABS) and acrylonitrile–styrene resin (AS) are two plastics widely used for the production of kitchen utensils because of their heat and impact resistance properties.

ABS and AS are manufactured from the monomers acrylonitrile, styrene, and/or 1,3-butadiene, and unreacted monomers have been detected in food contact materials made from these resins. Furthermore, other volatile substances such as benzene, toluene, 4-vinyl-1-cyclohexene, ethylbenzene, isopropylbenzene, and propylbenzene have also been detected in food contact materials made from these resins (Yoshida et al. 1979; Tan and Okada 1981; Gilbert and Startin 1982; Startin and Gilbert 1984; Tan et al. 1989; Ohno et al. 2002, 2008; Ohno and Kawamura 2010).

Some of these volatile substances have been identified as potentially carcinogenic or toxic. The International Agency for Research on Cancer (IARC) has classified 1,3-butadiene and benzene as group 1 carcinogens (carcinogenic to humans); styrene, acrylonitrile, ethylbenzene, 4-vinyl-1-cyclohexene, and isopropylbenzene as group 2B carcinogens (possibly carcinogenic to humans); methylmethacrylate and toluene as group 3 carcinogens (not classifiable as to their carcinogenicity to humans) (IARC 2013). These substances are possibly consumed with foods cooked using ABS or AS utensils and affect human health.

To ensure food safety, allowable levels of some of these volatile substances in food contact materials are regulated around the world. The U.S. Food and Drug Administration (FDA) specified maximum residual acrylonitrile levels in food contact materials made from ABS (11 *μ*g/g) and AS (50 or 80 *μ*g/g according to the degree of polymerization) in the Code of Federal Regulations (U.S. Food and Drug Administration 2013a,b). Meanwhile, the European Union (EU) established in a Commission Directive that the level of 1,3-butadiene in a finished product must be less than 1 *μ*g/g (European Commission 2011).

The Japanese Food Sanitation Law (FSL) (Ministry of Health, Labour and Welfare [MHLW] 2014) specifies a total maximum residual level of 5000 *μ*g/g for toluene, ethylbenzene, styrene, propylbenzene, and isopropylbenzene in kitchen utensils made from polystyrene (PS) and styrene-rich resins. However, resins with a styrene content of less than 50% such as ABS are exempt from the FSL. Furthermore, other volatile compounds such as 1,3-butadiene and acrylonitrile are not regulated in either ABS or AS. Japanese industries have, however, adopted a voluntary standard that requires the acrylonitrile level in finished products made from ABS and AS to be less than 80 *μ*g/g.

Previously, we determined the residual levels of volatile substances in ABS toys available in the Japanese market using a headspace gas chromatograph/mass spectrometer (HS-GC/MS) (Abe et al. 2013). In these toys, the residual levels of 14 volatile substances, including 1,3-butadiene, acrylonitrile, styrene, toluene, ethylbenzene, propylbenzene, isopropylbenzene, benzene, methylisobutyrate, methylmethacrylate, 4-vinyl-1-cyclohexene, 1-octene, xylene, and α-methylstyrene were determined. The residual levels of styrene and ethylbenzene were particularly high, with some samples containing >1000 *μ*g/g. These results suggest that the residual levels of these volatiles in Japanese ABS kitchen utensils may be high.

In this study, we surveyed both the residual levels of 14 volatile substances in ABS and AS kitchen utensils and the migration levels of these volatile substances into 20% ethanol using a HS-GC/MS.

## Experimental

### Samples

In this study, the samples labeled as ABS or AS products were used. Fifteen samples of ABS kitchen utensils (slicer, pick, peeler, lunch box, spoon, etc.) and 15 samples of AS kitchen utensils (cup, chopsticks, lunch box, plate, bowl, etc.) were purchased from Japanese markets in 2011. For the recovery tests, two ABS sheets (ABS sheet 1 and 2) and two AS sheets (AS sheet 1 and 2) were provided by The Japan Hygienic Olefin and Styrene Plastics Association.

### Reagents and standard solutions

*N,N*-Dimethylacetamide (DMA, high performance liquid chromatography grade) was purchased from Sigma-Aldrich Japan (Tokyo, Japan). Ethanol (for pesticide residue and polychlorinated biphenyl analysis, >99.5%) was purchased from Wako Pure Chemical Industries, Ltd. (Osaka, Japan).

The standards and internal standards used in this study are shown in Table 1. Mixed volatile substance standard stock solutions were prepared at concentrations ranged from 0.5 to 50,000 *μ*g/mL in DMA and ethanol. Mixed internal standard stock solutions were prepared as follows. For the analysis of the residual levels of volatile substances in ABS and AS kitchen utensils, 1,2-butadiene, isobutyronitrile, and *p*-diethylbenzene were mixed in DMA at 100, 10,000, and 1000 *μ*g/mL. Analysis of the migration levels of these volatile substances was performed using 20% ethanol solution containing the same concentrations.

**Table 1:** Standards and internal standards

					Monitor ion (*m/z*)	
					
Chemical name	CAS No.	Purity (%)	Supplier^1^	Retention time (min)	Quantifying ion	Qualifying ion
Standard						
1,3-Butadiene^2^	106-99-0	>95	A	6.1	54	39, 53
Acrylonitrile^2^	107-13-1	>99.8	B	10.7	53	52
Benzene	71-43-2	>99	C	13.2	78	52
Methyl isobutyrate	547-63-7	>95	D	13.6	43	71, 41
Methyl methacrylate	80-62-6	>99.8	E	14.1	69	41
1-Octene	111-66-0	>95	D	14.9	55	70
Toluene	108-88-3	>99.5	F	15.0	91	92
4-Vinyl-l-cyclohexene	100-40-3	>95	D	15.8	79	54, 91
Ethylbenzene	100-41-4	>99	D	16.3	91	106
*p-*Xylene	106-42-3	>98	D	16.4	91	106
Styrene	100-42-5	>99	D	16.8	104	78
Isopropylbenzene	98-82-8	>98	D	17.0	105	120, 91
Propylbenzene	103-65-1	>97	D	17.4	91	102, 105
α-Methylstyrene	98-83-9	>98	D	17.7	118	117, 91
Internal standard						
1,2-Butadiene^2^	590-19-2	>95	A	7.2	54	53, 39
Isobutyronitrile	78-67-1	>99	E	13.1	68	42
*p*-Diethylbenzene	105-05-5	>99	D	18.3	105	117, 120

1 A, Hayashi Pure Chemical Ind., Ltd.; B, AccuStandard; C, Kokusan Chemical Co., Ltd.; D, Wako pure Chemical Industries Ltd.; E, Tokyo Chemical Industry Co., Ltd.; F, Aldrich Chemical Co., Inc.

2 Methanol solution (1000 *μ*g/mL).

All standard and internal standard stock solutions were maintained in storage bottles with tightly sealed caps (Kanto Chemical Co. Inc., Tokyo, Japan) and stored at −20°C.

### HS-GC/MS conditions

The HS sampler (HP7694; Agilent Technologies, Santa Clara, CA) conditions were as follows. For the determination of residual levels of volatile substances, the oven temperature was held at 90°C for 60 min. The sample loop temperature was at 110°C and transfer-line temperature was 150°C. The injection time was 0.5 min and injection volume was 1.0 mL. Both the sample loop filling time and the sample loop equilibration time were 0.1 min. For the determination of migration levels of volatile substances, the oven temperature was held at 60°C for 30 min. The sample loop temperature was at 80°C and transfer-line temperature was 200°C. The other parameters were same as mentioned above.

The GC/MS (6890 GC and 5973 MSD; Agilent Technologies) conditions were as follows. A DB-624 (0.25 mm i.d. × 60 m, 1.4 *μ*m film thickness; Agilent Technologies) column was used. The oven temperature was initially held at 40°C for 7 min, then increased at 20°C/min to 250°C and then held at that temperature for 5 min. The injection temperature was 200°C and the transfer-line temperature was 250°C. The helium carrier gas flow rate was constant at 1.2 mL/min. A split injection mode was used with a ratio of 10:1, and the ion source voltage was 70 eV. Finally, the selected ion monitoring (SIM) mode was used, and the monitored ions are given in Table 1.

### Test methods

#### Residual volatile substances

The residual levels of volatile substances in ABS and AS kitchen utensils were determined using a previously reported method (Abe et al. 2013). Each sample (0.5 g) was cut into small pieces and then placed along with 5 *μ*L of a mixed internal standard stock solution in DMA and 2.5 mL of DMA in a 20-mL HS vial, which was immediately tightly sealed. The vial was stored overnight at room temperature and the sample was analyzed via HS-GC/MS after it completely dissolved.

#### Migration of volatile substances

To determine the migration levels of the volatile substances into 20% ethanol, migration tests were performed according to the condition of Japanese official method in the FSL (MHLW). The 20% ethanol (2 mL/cm^2^) was added to a cut sample and the sample was incubated at 60°C for 30 min. After the incubation, 2 mL of the solution was transferred to a 20-mL HS vial containing 5 *μ*L of the mixed internal standard stock solution in ethanol. The vial was immediately tightly sealed and HS-GC/MS analysis was performed.

### Calibration curves and limits of quantification

To construct calibration curves for determination of the residual levels and migration quantities of the volatile substances, standard solutions were prepared as follows.

For determination of the residual levels of the volatile substances in the ABS and AS kitchen utensils, 5–50 *μ*L quantities of the mixed standard stock solutions in DMA (0.5–50,000 *μ*g/mL) were added to DMA (2.5 mL) in 20-mL HS vials in order to obtain the desired concentrations (0.001–500 *μ*g/mL). Subsequently, 5 *μ*L of mixed internal standard stock solution in DMA was added to each vial and the vials were immediately tightly sealed.

For determination of the quantities of the volatile substances that migrated from the ABS and AS kitchen utensils, 4–20 *μ*L of mixed standard stock solutions in ethanol were added to water (2.0 mL) in 20-mL HS vials in order to obtain the desired concentrations (1–50 ng/mL). Subsequently, 5 *μ*L of mixed internal standard stock solution in ethanol was added, and the vials were immediately tightly sealed.

The prepared standard solutions in the HS vials were analyzed using HS-GC/MS, and calibration curves were constructed by plotting the peak area ratios for 1,3-butadiene versus 1,2-butadiene, for acrylonitrile versus isobutyronitrile, and for the other volatile substances versus *p*-diethylbenzene.

The limit of quantification (LOQ) was defined for all of the volatile substances as a signal-to-noise ratio (S/N) of 10/1 for the peak intensity.

### Recovery test

To evaluate the accuracy of the method for determining residual volatile substances in the ABS and AS samples, recovery tests were performed. The mixed standard stock solutions were spiked with two ABS sheets and two AS sheets. The analytes other than styrene and ethylbenzene were spiked at levels equivalent to 0.5 and 2 *μ*g/g. Styrene and ethylbenzene were spiked at levels equivalent to 50 and 250 *μ*g/g because the residual levels of styrene and ethylbenzene in the sheets were high (approximately 50–2000 *μ*g/g).

## Results and Discussion

### Assessment of the method for the determination of residual volatiles

Previously, we developed a method for the simultaneous determination of the residual levels of various volatile substances in ABS toys and assessed the linearity, accuracy, and precision of this method (Abe et al. 2013). In this study, the application of this method to determine the residual levels of volatiles in ABS and AS kitchen utensils was investigated. To assess the linearity of the method, calibration curves for 14 volatile substances were constructed using DMA as the sample solvent. Good linearity was achieved over the concentration ranges 0.005–10 *μ*g/mL for 1,3-butadiene and acrylonitrile, 0.025–500 *μ*g/mL for isopropylbenzene, and 0.01–500 *μ*g/mL for the other volatile substances. The correlation coefficients (*R*^2^) for all of the volatile substances were >0.998. The LOQs for the residual volatile substances in the ABS and AS kitchen utensils were estimated to be 0.025 *μ*g/g for 1,3-butadiene and acrylonitrile, 0.13 *μ*g/g for isopropylbenzene, and 0.05 *μ*g/g for the other volatile substances.

To assess the accuracy and precision of this method, recovery tests were performed in triplicate (Table 2). The recovery rates for the volatile substances found in ABS and AS sheets ranged from 88% to 108%, and the relative standard deviation (RSD) values were in the range from 1% to 6%. Because the residual levels of methylmethacrylate and 4-vinyl-1-cyclohexene in ABS sheet 1 were greater than the 0.5 or 2 *μ*g/g spiking levels, these recoveries and RSD values were not tested. However, when these volatiles were spiked at concentrations of 50 and 250 *μ*g/g, recovery rates and RSD values were all in the range from 97% to 104% and from 1% to 4%, respectively. Consequently, acceptable linearity, recovery rates, and RSD values were obtained for all of the substances, suggesting that this method is reliable for the quantitative determination of the amounts of volatile substances in ABS and AS kitchen utensils.

**Table 2:** Recovery rates of 14 volatile substances in ABS and AS sheets

		ABS		AS	
			
Substance	Spiked levels (*μ*g/g)	Sheet 1	Sheet 2	Sheet 1	Sheet 2
1,3-Butadiene	0.5	101 ± 1	101 ± 3	99 ± 2	102 ± 3
	2	103 ± 2	104 ± 2	103 ± 1	103 ± 3
Acrylonitrile	0.5	98 ± 4	102 ± 4	98 ± 4	102 ± 4
	2	102 ± 2	100 ± 3	102 ± 2	100 ± 3
Benzene	0.5	104 ± 4	96 ± 5	95 ± 1	92 ± 1
	2	101 ± 2	96 ± 1	97 ± 1	105 ± 6
Methyl isobutyrate	0.5	100 ± 1	101 ± 2	101 ± 2	95 ± 3
	2	101 ± 1	98 ± 1	99 ± 1	105 ± 3
Methyl methacrylate	0.5	—	101 ± 1	95 ± 4	96 ± 1
	2	—	100 ± 1	98 ± 1	105 ± 3
1-Octene	0.5	102 ± 4	95 ± 5	96 ± 3	91 ± 2
	2	99 ± 2	97 ± 1	101 ± 3	105 ± 3
Toluene	0.5	97 ± 5	90 ± 3	104 ± 5	94 ± 3
	2	104 ± 1	103 ± 1	97 ± 1	104 ± 5
4-Vinyl-1-cyclohexene	0.5	—	99 ± 2	97 ± 1	93 ± 3
	2	—	102 ± 4	96 ± 1	105 ± 6
Ethylbenzene	50	101 ± 1	96 ± 2	101 ± 3	101 ± 2
	250	99 ± 1	99 ± 1	99 ± 1	101 ± 1
*p-*Xylene	0.5	102 ± 2	98 ± 1	100 ± 1	102 ± 4
	2	103 ± 1	103 ± 1	101 ± 1	98 ± 2
Styrene	50	103 ± 1	98 ± 1	103 ± 1	103 ± 2
	250	103 ± 3	103 ± 2	99 ± 4	105 ± 2
Isopropylbenzene	0.5	96 ± 6	88 ± 4	97 ± 4	102 ± 4
	2	101 ± 2	108 ± 3	104 ± 2	102 ± 1
Propylbenzene	0.5	99 ± 6	95 ± 1	94 ± 3	97 ± 4
	2	97 ± 2	100 ± 3	104 ± 2	101 ± 4
α-Methylstyrene	0.5	103 ± 5	101 ± 2	102 ± 2	99 ± 1
	2	101 ± 3	100 ± 2	99 ± 2	103 ± 5

Mean ± SD of three trials (%). ABS, acrylonitrile–butadiene–styrene resin; AS, acrylonitrile–styrene resin.

### Residual levels of volatile substances in ABS kitchen utensils

Residual levels of the volatile substances in 15 ABS kitchen utensils are shown in Table 3. Eleven volatile substances, including 1,3-butadiene, acrylonitrile, and benzene, were detected in all of the kitchen utensil samples examined in this study. Notably, the residual levels of ethylbenzene and styrene were in the range from 30 to 960 and 110 to 2000 *μ*g/g, respectively, and were significantly higher than those of the other detected substances. In addition, methylmethacrylate, 4-vinyl-1-cyclohexene, and α-methylstyrene were present at reasonably high concentrations of 0.23–250, 0.16–320, and 0.28–100 *μ*g/g, respectively. The residual levels of the remaining volatile substances were <50 *μ*g/g. These results indicate that the residual levels of these 14 volatile substances in the 15 kitchen utensils made from ABS are similar to those in the previously evaluated ABS toys (Abe et al. 2013).

**Table 3:** Residual levels of 14 volatile substances in ABS kitchen utensils

Sample	1,3-BD	AN	BZ	MIB	MMA	1OC	TO	4-VC	EB	XYL	ST	IPB	PB	α-MST
ABS1	0.59	4.4	0.10	NQ	NQ	0.49	0.34	2.0	85	1.4	120	2.7	1.7	6.5
ABS2	0.69	3.8	0.07	NQ	NQ	0.34	0.29	1.5	75	0.78	110	2.8	1.5	4.5
ABS3	0.10	15	0.28	NQ	0.23	NQ	0.74	49	180	11	750	22	7.6	6.9
ABS4	0.39	12	0.64	NQ	1.8	NQ	1.1	22	260	13	920	30	20	1.2
ABS5	0.97	0.55	0.69	4.2	200	0.37	2.6	1.8	750	28	330	13	6.7	0.35
ABS6	1.2	1.8	0.79	8.1	250	0.30	3.3	1.4	960	17	440	15	10	0.28
ABS7	0.20	20	0.05	NQ	18	NQ	0.40	21	70	29	990	18	7.1	46
ABS8	0.21	14	0.05	NQ	21	NQ	0.24	10	83	9.8	800	15	7.5	97
ABS9	0.36	7.6	0.11	NQ	NQ	0.21	0.60	7.7	190	3.1	460	9.9	4.0	68
ABS10	0.79	6.9	0.20	NQ	NQ	0.81	4.9	5.6	160	2.0	210	8.3	2.8	16
ABS11	0.73	13	0.12	NQ	NQ	NQ	0.77	66	30	1.9	660	14	3.0	100
ABS12	1.3	0.71	0.36	7.0	200	0.30	2.8	2.2	630	10	290	10	7.7	0.40
ABS13	1.7	11	0.12	NQ	NQ	1.5	1.9	320	46	4.2	2000	31	4.8	17
ABS14	0.06	0.15	0.12	NQ	0.60	0.49	2.3	0.16	120	6.6	350	23	19	0.98
ABS15	0.62	13	0.84	NQ	NQ	NQ	1.6	28	260	12	900	27	19	0.68
LOQ	0.025	0.025	0.05	0.05	0.05	0.05	0.05	0.05	0.05	0.05	0.05	0.13	0.05	0.05

Mean of two trials (*μ*g/g), NQ = not quantified (Under the LOQ). ABS, acrylonitrile–butadiene–styrene resin.

The total residual levels of five volatile substances (toluene, ethylbenzene, styrene, isopropylbenzene, and propylbenzene) regulated in Japan for PS kitchen utensils ranged from 190 to 2100 *μ*g/g (average: 910 *μ*g/g). These levels were less than the regulatory limit for PS kitchen utensils as established in the Japanese FSL (5000 *μ*g/g). However, the residual levels of 1,3-butadiene detected in three of the 15 samples exceeded the EU regulatory limit (1 mg/kg) and those of acrylonitrile in seven of the 15 samples exceeded the U.S. FDA limit (11 *μ*g/g).

Historically, the residual levels of acrylonitrile in ABS kitchen utensils in Japan were 0–138 *μ*g/g (Yoshida et al. 1979) and 0.3–50.4 *μ*g/g (Ohno and Kawamura 2010). The present results are similar to the results reported in 2010. In addition, the total residual level of the five volatile substances regulated for PS kitchen utensils in Japan ranged from 100 to 10,320 *μ*g/g (average: 1900 *μ*g/g) in 1979. Thus, the total residual levels of these five volatile substances in this study were significantly lower than those reported in 1979.

### Residual levels of volatile substances in AS kitchen utensils

Residual levels of the volatile substances in 15 AS kitchen utensils are shown in Table 4. Seven volatile substances, including acrylonitrile, toluene, and ethylbenzene, were detected in all 15 samples and benzene and α-methylstyrene were detected in some of the samples. The 1,3-butadiene, methylisobutyrate, methylmethacrylate, 1-octene, and 4-vinyl-1-cyclohexene were not detected in any of the AS kitchen utensils. However, residual levels of ethylbenzene and styrene ranged from 76 to 1000 and 430 to 2800 *μ*g/g, respectively, which were significantly higher than those of the others substances, followed by toluene and acrylonitrile with concentrations ranging from 0.43 to 570 and 20 to 180 *μ*g/g, respectively. The residual levels of the remaining volatile substances were <50 *μ*g/g. These results suggest that the residual levels of acrylonitrile, ethylbenzene, and styrene in AS kitchen utensils are greater than those in ABS kitchen utensils, whereas those of α-methylstyrene in kitchen utensils made from AS are lower than those in kitchen utensils made from ABS.

**Table 4:** Residual levels of 14 volatile substances in AS kitchen utensils

Sample	1,3-BD	AN	BZ	MIB	MMA	1-OC	TO	4-VC	EB	XYL	ST	IPB	PB	α-MST
AS1	NQ	160	0.61	NQ	NQ	NQ	570	NQ	120	12	2000	26	18	0.09
AS2	NQ	28	0.36	NQ	NQ	NQ	1.4	NQ	1000	6.4	720	17	12	0.23
AS3	NQ	23	0.36	NQ	NQ	NQ	1.5	NQ	1000	5.8	670	15	12	0.16
AS4	NQ	23	NQ	NQ	NQ	NQ	0.51	NQ	400	4.1	570	15	12	ND
AS5	NQ	19	NQ	NQ	NQ	NQ	0.78	NQ	76	0.32	1900	6.3	4.3	0.57
AS6	NQ	47	NQ	NQ	NQ	NQ	0.57	NQ	200	21	1100	15	18	0.44
AS7	NQ	54	0.22	NQ	NQ	NQ	1.3	NQ	590	6.4	520	7.9	6.0	0.27
AS8	NQ	21	0.33	NQ	NQ	NQ	1.5	NQ	800	4.8	690	14	10	NQ
AS9	NQ	54	0.25	NQ	NQ	NQ	2.8	NQ	640	4.8	600	10	7.5	0.14
AS10	NQ	20	0.34	NQ	NQ	NQ	1.4	NQ	1000	4.9	700	8.4	7.4	0.31
AS11	NQ	43	NQ	NQ	NQ	NQ	0.43	NQ	180	16	990	13	15	0.28
AS12	NQ	55	0.27	NQ	NQ	NQ	1.2	NQ	650	4.9	630	11	7.6	0.33
AS13	NQ	180	0.53	NQ	NQ	NQ	0.80	NQ	250	11	2800	68	46	1.3
AS14	NQ	150	0.54	NQ	NQ	NQ	1.5	NQ	200	13	2700	57	40	0.73
AS15	NQ	21	NQ	NQ	NQ	NQ	0.89	NQ	450	3.5	430	5.5	3.2	NQ
LOQ	0.025	0.025	0.05	0.05	0.05	0.05	0.05	0.05	0.05	0.05	0.05	0.13	0.05	0.05

Mean of two trials (*μ*g/g), NQ = not quantified (Under the LOQ). AS, acrylonitrile–styrene resin.

The total residual levels of five volatile substances (toluene, ethylbenzene, styrene, isopropylbenzene, and propylbenzene) regulated in Japan for PS kitchen utensils were 830–3200 *μ*g/g (average: 1700 *μ*g/g) and fell below the regulatory limit for volatile substances in PS kitchen utensils as established in the Japanese FSL (5000 *μ*g/g). However, the residual levels of acrylonitrile in three out of the 15 AS samples exceeded the voluntary standard set by Japanese industries (80 *μ*g/g). Moreover, those in six and three out of the 15 AS samples exceeded the U.S. FDA limits (50 and 80 *μ*g/g, respectively).

Yoshida et al. (1979) reported that the residual levels of acrylonitrile in 79 AS kitchen utensils obtained from Japanese markets ranged from 4 to 376 *μ*g/g, and Ohno and Kawamura (2010) reported that those in five Japanese AS kitchen utensils ranged from 16.8 to 54.5 *μ*g/g. The present results are thus similar to those reported in 2010. In 1979, total residual levels of the five volatile substances regulated for PS kitchen utensils in Japan ranged from 560 to 7390 *μ*g/g (average: 2600 *μ*g/g). Clearly, the total residual levels of these five volatile substances obtained in this study are significantly lower than those reported in 1979.

Differences in the residual levels of volatile substances found in 1979 and this study for ABS and AS kitchen utensils can be attributed to an improvement in the quality of the materials due to more complete polymerization, which has been achieved through advances in manufacturing technology and the adoption of increasingly strict EU regulations.

### Migration levels from ABS and AS kitchen utensils

To determine migration levels of volatile substances from ABS and AS kitchen utensils, migration tests were performed using a food simulant. ABS and AS kitchen utensils are typically used for common foods, while they are occasionally used for fatty foods. In the case of common foods, water is used as the simulant in Japan, whereas 10% ethanol is used in the U.S. and EU. For more effective migration of fat-soluble substances into the solvent, thus the migration tests were performed using 20% ethanol. The migration tests were performed at 60°C for 30 min. This condition refers to the official method of plastic migration test in Japanese FSL (MHLW).

To assess the linearity of determining the migration of volatile substances, calibration curves were constructed using 20% ethanol as the sample solvent. Good linearity was achieved over the concentration range from 3 to 50 ng/mL for all of the volatile substances, and *R*^2^ was >0.994 for all of them. The LOQs for the quantities of the volatile substances that migrated into 20% ethanol from ABS and AS kitchen utensils were estimated to be 6 ng/cm^2^ for all of the volatile substances.

As shown in Table 5, migration levels of the volatiles in 10 of the 15 ABS samples and 10 of the AS samples into 20% ethanol at 60°C for 30 min were determined using HS-GC/MS. Methylmethacrylate, ethylbenzene, xylene, and styrene from the ABS samples and acrylonitrile, ethylbenzene, and styrene from the AS samples were detected in the simulant. Styrene was detected in the simulant for all but two ABS samples because of its high residual levels. The quantities of volatile substances that migrated into the simulant ranged from 6 to 76 ng/cm^2^.

**Table 5:** Migration amounts of volatile substances from ABS and AS kitchen utensils

Sample	MMA	EB	XYL	ST	Sample	AN	EB	ST
ABS1	NQ	NQ	NQ	NQ	AS1	18	NQ	46
ABS2	NQ	NQ	NQ	NQ	AS2	NQ	12	16
ABS4	NQ	NQ	NQ	34	AS3	NQ	14	16
ABS6	16	12	NQ	12	AS4	NQ	8	10
ABS7	NQ	NQ	NQ	8	AS5	NQ	NQ	32
ABS8	NQ	NQ	NQ	30	AS6	NQ	NQ	22
ABS9	NQ	6	6	16	AS7	NQ	8	12
ABS11	NQ	NQ	46	40	AS8	NQ	14	18
ABS12	16	22	30	16	AS12	NQ	6	10
ABS13	NQ	NQ	NQ	76	AS15	NQ	NQ	8
LOQ	6	6	6	6		6	6	6

Mean of two trials (ng/cm^2^), NQ = not quantified (Under the LOQ). ABS, acrylonitrile–butadiene–styrene resin; AS, acrylonitrile–styrene resin.

**Figure 1:**
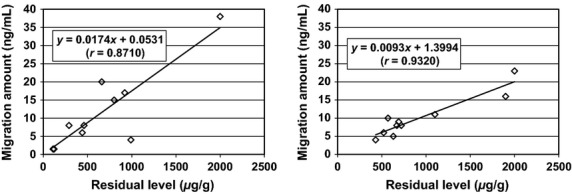
Correlations between residual levels and migration levels of styrene in ABS (left) and AS (right) kitchen utensils. The equation in graphs shows a regression line equation and “*r*” in parentheses shows the correlation coefficient of the regression line. ABS, acrylonitrile–butadiene–styrene resin; AS, acrylonitrile–styrene resin.

The migration levels of styrene correlated well with the residual levels (*r* = 0.8710 in ABS kitchen utensils and 0.9320 in AS kitchen utensils, Fig. 1). The slope of the ABS regression line (0.0174) was greater than that of the AS regression line (0.0093). In addition, xylene migrated only from the ABS samples. These results suggest that the migration levels for styrene and xylene from the ABS samples were higher than those from the AS samples, which might be attributed to the softness of ABS.

It should be noted that the European Standard suggests that volatile substances can readily migrate from the cut edges of ABS and AS samples (European Standard EN 13130-1:2004, 2004). Therefore, the accurate assessment of the migration of volatile substances from the surfaces of kitchen utensils, other than from cut edges, is difficult; thus, the actual migration level into foods might be lower than those reported in this study.

Acrylonitrile, styrene, and ethylbenzene, which were detected in the above described migration tests, are classified as group 2B carcinogens by IARC. Although it is possible that these compounds migrate from kitchen utensils into foods, there currently are no regulations for 1,3-butadiene and styrene in the Japan FSL. Regulatory limits for these volatiles are therefore required in Japan in order to ensure the safety of food contact materials.

## Conclusion

Residual levels of 14 volatile substances in ABS and AS kitchen utensils in Japan were determined using HS-GC/MS. All 14 volatile substances were detected in the ABS kitchen utensils and nine volatile substances were found in the AS samples. Although the residual levels of acrylonitrile and 1,3-butadiene were lower than those reported over 30 years ago, those residual levels exceeded the Japanese voluntary standard and the regulatory limits established in the U.S. and EU in some kitchen utensils. In addition, the residual levels of ethylbenzene and styrene were found to be >1000 *μ*g/g in some samples. Therefore, the results of this study are very important for ensuring the safety of food contact materials made from ABS and AS and indicate that further study is needed.
